# Evaluation of the analytical performances of the Cobas c513 analyser for HbA_1c_ assay

**DOI:** 10.11613/BM.2018.030708

**Published:** 2018-10-15

**Authors:** Stéphane Jaisson, Nathalie Leroy, Michel Soulard, Aurore Desmons, Emmanuelle Guillard, Philippe Gillery

**Affiliations:** 1 Biochemistry Department, University Hospital of Reims, Reims, France; 2 Bio Paris Ouest Laboratory, Levallois, France

**Keywords:** diabetes, evaluation, HbA_1c_, immunoassay

## Abstract

**Introduction:**

Haemoglobin A_1c_ (HbA_1c_) is considered to be the gold standard for the follow-up of glycaemic control in patients with diabetes mellitus and is also a diagnostic tool. Accordingly, reliable and efficient methods must be used for its quantification. Roche Diagnostics have recently adapted the Tina-quant® HbA_1c_ Third Generation immunoassay on a fully dedicated analyser, the Cobas c513, which allows a high throughput of up to 400 samples *per* hour. The present article deals with the evaluation of the analytical performances of this system which has been recently introduced to the market.

**Materials and methods:**

Precision, comparison with two ion-exchange high-performance liquid chromatography (HPLC) methods (Variant II and D-100 systems, BioRad Laboratories) using Passing Bablok and Bland-Altman analyses, accuracy and interference of the most frequent haemoglobin (Hb) variants on HbA_1c_ measurement were evaluated.

**Results:**

Precision was high, with coefficients of variation lower than 1.1% (HbA_1c_ values expressed in National Glycohemoglobin Standardization Program units, 1.7% for values expressed in International Federation of Clinical Chemistry and Laboratory Medicine [IFCC] units). The comparison study showed similar results with the two HPLC systems. The analysis of samples with IFCC-assigned values showed high methodological accuracy. Finally, no interference of bilirubin, triglycerides and common Hb variants (Hb AC, AD, AE, AS) was observed.

**Conclusions:**

This evaluation showed that the analytical performance of the Cobas c513 analyser for HbA_1c_ assay makes it suitable for a routine use in clinical chemistry laboratories.

## Introduction

The term haemoglobin A_1c_ (HbA_1c_) is commonly used to describe the major fraction of glycated haemoglobin in blood, which results from the non-enzymatic binding of glucose on the N-terminal valine residues of haemoglobin β chains. Haemoglobin A_1c_ is considered the best marker for the monitoring of diabetic patients because its evaluation provides retrospective information on the glycaemic balance of patients for the past 6-8 weeks (*1*). Moreover, elevated HbA_1c_ values are associated with the development of long-term complications in type 1 and type 2 diabetes (*2*-*4*). The number of countries recommending the use of HbA_1c_ for the diagnosis of diabetes, with a threshold value of 48 mmol/mol (6.5%), has risen in recent years (*5*, *6*). The use of HbA_1c_ for such diagnostic purposes requires reliable, accurate, and robust analytical methods in order to avoid misdiagnosis, which could result in high unwarranted expenditure on patient care in the case of over-diagnosis. Consequently, available diagnostic methods must be traceable to internationally accepted reference methods in order to improve the overall comparability of methods and assessment of their quality performance (*7*, *8*).

The evaluation of HbA_1c_ values may be performed using a number of methods based on various principles, ranging from separative methods (*e.g.* ion-exchange chromatography and capillary electrophoresis) to immunological assays. Due to both, the increasing number of patients with diabetes worldwide and the expanded use of HbA_1c_ for diabetes diagnosis, the need for HbA_1c_ assessment is constantly increasing. To meet these needs, manufacturers are now offering robust analytical solutions with higher throughput than previously available, particularly in laboratory settings.

Roche Diagnostics have recently adapted the Tina-quant® HbA_1c_ Third Generation immunoassay on a fully dedicated analyser, the Cobas c513, which allows a sample throughput of up to 400 samples *per* hour. The present study was performed to evaluate the analytical performance of this system, with a focus on comparison with two routine high-performance liquid chromatography (HPLC) systems (D-100 and Variant II analysers from Bio-Rad Laboratories), the accuracy and precision of the method, the interference of the most frequent haemoglobin (Hb) variants, and the usability of the system.

## Materials and methods

### Materials

The analyser and reagents used for this evaluation were provided by Roche Diagnostics (Mannheim, Germany) and were used according to manufacturer’s instructions.

#### Cobas c513 analyser

Cobas c 513 is a fully dedicated HbA_1c_ assay analyser with a high throughput, owing the use of ready-to-use reagents in a large kit size. This analyser also makes use of minimized operator intervention; from sample registration to result delivery, and a closed-tube sampling function. On-board stability of the reagents is 4 weeks. The analyser must be calibrated every month or at each change of the reagent lot number.

HbA_1c_ determination utilizes the Tina-quant® Third Generation assay, which is based on a turbidimetric inhibition immunoassay (TINIA) for haemolysed whole blood. The first step is the preparation of the blood haemolysate using a detergent-containing reagent. The second step is the spectrophotometric measurement of total Hb in the haemolysate, converted to a stable derivative at 376 nm. In parallel, HbA_1c_ reacts with the anti-HbA_1c_ antibody to form soluble antigen-antibody complexes. Finally, the polyhaptens contained in the reagent react with excess anti-HbA_1c_ antibodies and form an insoluble antibody-polyhapten complex, which is measured by turbidimetry at 340 nm (the higher the HbA_1c_ concentration, the lower the turbidity). The output of this method is linear from 23 mmol/mol (4.3%) to 196 mmol/mol (20.1%).

#### Samples

For the comparison study, blood samples collected in ethylenediaminetetraacetic acid (EDTA)-containing tubes (Sarstedt, Nümbrecht, Germany) received by the Bio-Paris-Ouest laboratory for HbA_1c_ measurement, were selected following analysis with the D-100 system. Samples were then shipped to the Reims University Hospital laboratory at 2-8 °C within the 48 hours, for HbA_1c_ measurement with the Cobas c513 system.

For the precision study, blood samples were collected in EDTA-containing tubes (BD Vacutainer, Le Pont de Claix, France) and sent to the laboratory for routine HbA_1c_ assay. No additional samples were necessary for this study, and no samples were stored after the assays. Quality control (QC) samples (PreciControlA1c Norm and PreciControlA1c Path), blood samples with International Federation of Clinical Chemistry and Laboratory Medicine (IFCC)-assigned values and blood samples with Hb variants were provided by Roche Diagnostics.

### Methods

#### Precision study

The precision study was performed according to Clinical & Laboratory Standards Institute (CLSI) EP05-A3 protocol (*9*). Quality controls (two levels) and patient samples (four levels covering the analytical range) were analysed in duplicate, twice daily for 21 days (*i.e.* 84 determinations). Samples were aliquoted and stored at - 80 °C to avoid freezing/thawing cycles. The analyser was calibrated twice during the precision study and the same reagent and calibrator lot numbers were used. The acceptance criteria for precision were total imprecision coefficients of variation (CVs) lower than 2% as recommended in the literature (*6*).

#### Comparison study

The comparison study was performed according to an internal protocol. For that purpose, samples (N = 100) were assayed using Cobas c513 and two routinely used HPLC analysers (Variant II equipped with a Dual Kit and D-100, Bio-Rad Laboratories, Hercules, USA). Haemoglobin A_1c_ values were compared by Passing Bablok regression and Bland-Altman analyses, using samples with HbA_1c_ values distributed across the linearity range (*10*, *11*). Samples with Hb variants or elevated values of carbamylated Hb (cHb) or labile HbA_1c_ were excluded from the comparison study. All comparative assays were performed within a 48 hour-period, and samples were kept at + 4 °C until analysis. The interpretation of data was based on the results of Passing Bablok regression analysis, which allows the determination of constant and proportional errors, and Bland-Altman analysis in which the proportion of outliers had to be lower than 5%.

#### Accuracy

Eight external assurance quality samples with IFCC-assigned values obtained from the European Reference Laboratory for Glycohemoglobin (Winterswijk, The Netherlands) were analysed in triplicate using the Cobas c513 analyser. Absolute and relative biases between the target and the obtained values were calculated to evaluate the accuracy of the method. The acceptance criteria for accuracy were based on those used for the sigma-metrics model calculation, in which absolute biases should be lower than 5 mmol/mol in IFCC units (*12*).

#### Interferences

The influence of bilirubin and triglycerides on HbA_1c_ quantification was studied by mixing washed red blood cells with various dilutions of hyperbilirubinemic or triglyceride-rich plasmas to obtain bilirubin and triglyceride concentrations reaching 352 μmol/L and 20.6 mmol/L, respectively.

The influence of the most common Hb variants (Hb AC, AD, AE, AS) on HbA_1c_ measurement was determined by analysing samples containing Hb variants (N = 10 *per* variant) with various concentrations of HbA_1c_. Each sample had assigned target values using a comparison method (IFCC calibrated boronate affinity chromatography method, Premier Hb9210) which is known to be unaffected by the presence of Hb variants (assignment performed at the European Reference Laboratory for Glycohemoglobin, The Netherlands). In parallel, 40 HbAA samples covering the same HbA_1c_ range as variant samples were assayed in order to assess the relative deviation of variant samples compared to normal ones. Haemoglobin AA results were used for the calculation of a regression line, any difference exceeding ± 10% with respect to this line in Hb variant samples was considered clinically significant (*13*).

#### Influence of red blood cell sedimentation

In order to determine the impact of red blood cell sedimentation on the measurement of HbA_1c_, ten samples were assayed after 1h, 2h, 5h and 24h incubation periods at 4 °C or at room temperature. Tubes were not shaken during this incubation time so as to not disturb the blood cell sedimentation process. After the 24h incubation time, all samples were homogenized and analysed again. Values were compared with HbA_1c_ values obtained on agitated samples before starting the incubation period. The acceptance criteria were based on relative biases which had to be close to zero and a mean relative bias lower than 5%.

### Statistical analyses

Statistics were performed using Graph-Pad Prim 6.0 and R softwares.

## Results

### Precision study

For QC samples, the CVs were lower than 1.1% when HbA_1c_ values were expressed in % (National Glycohemoglobin Standardization Program [NGSP] units) and lower than 1.4% when values were expressed in mmol/mol (IFCC units) (Table 1). For patient samples, CVs were lower than 1.1% and 1.7% when HbA_1c_ values were expressed in NGSP and in IFCC units, respectively, irrespective of the level of HbA_1c_.

**Table 1:** Results of the precision study on the Cobas c513

	**Coefficients of variation (%)**							
**Sample**	**Mean value**		**Repeatability**		**Between-run**		**Between-day**	**Intermediate precision (total)**
**HbA_1c_, %**								
**Sample 1**		5.8		0.5		0.6	0.8	1.1
**Sample 2**		6.1		0.7		0.6	0.7	1.1
**Sample 3**		7.9		0.5		0.8	0.6	1.1
**Sample 4**		11.6		0.6		0.6	0.7	1.1
**QC sample (low-level)**		5.7		0.6		0.5	0.4	0.84
**QC sample (high-level)**		11.1		0.5		0.2	0.9	1.1
**HbA_1c_, mmol/mol**								
**Sample 1**		39.9		0.7		1.0	1.2	1.7
**Sample 2**		42.9		1.0		1.0	1.0	1.7
**Sample 3**		62.9		0.7		1.1	0.9	1.6
**Sample 4**		103.5		0.8		0.8	0.9	1.4
**QC sample (low-level)**		39.2		0.9		0.7	0.7	1.3
**QC sample (high-level)**		98.2		0.7		0.3	1.1	1.4
Acceptance criteria: the analytical goals usually admitted for precision performances of HbA_1c_ methods are intermediate precision CVs lower than 2% (*6*). HbA_1c_ - haemoglobin A_1c_. QC - quality control.								

### Accuracy

The relative biases ranged from - 0.2% to + 3.4%; the mean relative bias was + 1.6% for HbA_1c_ values expressed in IFCC units (+ 1.2% for values expressed in NGSP units) (Table 2). All absolute biases were lower than 3 mmol/mol and the mean absolute bias in IFCC units was + 1.1 mmol/mol (+ 0.10% HbA_1c_ in NGSP units).

**Table 2:** Comparison of HbA_1c_ results obtained using Cobas c513 on eight external assurance quality samples with IFCC-assigned values

	**HbA_1c_, mmol/mol**			
**EAQ sample**	**IFCC target value**	**Measured value**	**Absolute bias (mmol/mol)**	**Relative bias (%)**
**Sample 1**	31.4	32.1	+ 0.7	+ 2.2
**Sample 2**	38.7	38.9	+ 0.2	+ 0.5
**Sample 3**	49.6	49.5	- 0.1	- 0.2
**Sample 4**	58.5	59.3	+ 0.8	+ 1.2
**Sample 5**	69.0	69.8	+ 0.8	+ 1.2
**Sample 6**	78.3	80.6	+ 2.3	+ 2.9
**Sample 7**	88.6	91.6	+ 3.0	+ 3.4
**Sample 8**	99.2	100.4	+ 1.2	+ 1.2
		**Mean**	+ 1.1	+ 1.6
	**HbA_1c_, %**			
**Sample 1**	5.0	5.1	+ 0.07	+ 1.5
**Sample 2**	5.7	5.7	+ 0.02	+ 0.3
**Sample 3**	6.7	6.7	- 0.01	- 0.1
**Sample 4**	7.5	7.6	+ 0.08	+ 1.0
**Sample 5**	8.4	8.5	+ 0.07	+ 0.9
**Sample 6**	9.3	9.5	+ 0.21	+ 2.3
**Sample 7**	10.3	10.5	+ 0.27	+ 2.6
**Sample 8**	11.2	11.3	+ 0.11	+ 0.9
		**Mean**	+ 0.10	+ 1.2
EAQ - external assurance quality. The measured value represents the mean of three HbA_1c_ determinations. The acceptance criterion for the evaluation of accuracy is absolute biases lower than 5 mmol/mol in IFCC units (*12*). HbA_1c_ - haemoglobin A_1c_. IFCC - International Federation of Clinical Chemistry and Laboratory Medicine.				

### Comparison study

**Figure 1:**
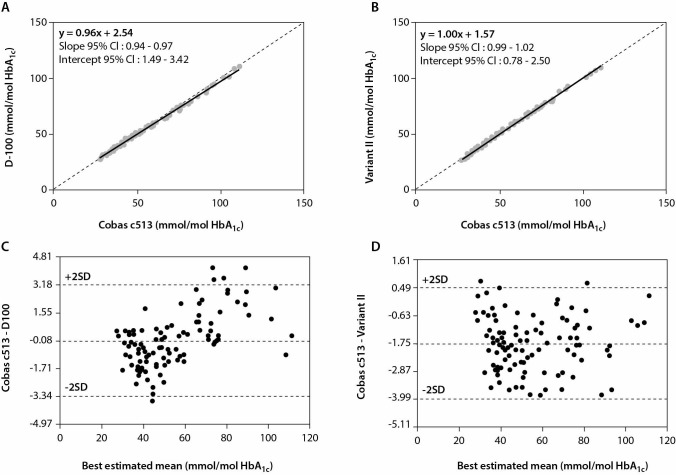
Comparison of HbA_1c_ values obtained with Cobas c513 and D-100^TM^ or Variant^TM^ II systems (N = 100). The methods have been compared using Passing-Bablock regression (A and B) and Bland-Altman analyses (C and D). HbA_1c_ - haemoglobin A_1c_.

Haemoglobin A_1c_ values obtained with Cobas c513 were compared with those obtained with two other routine analysers: D-100 and Variant II. The comparison with D-100 system showed good results, with the following linear regression equation: y (HbA_1c_ D-100, mmol/mol) = 0.96 x (HbA_1c_ Cobas c513, mmol/mol) + 2.54, 95% confidence intervals being comprised between 0.94 and 0.97 for slope and 1.49 and 3.42 for intercept (Figure 1A). These results indicated slight constant and proportional errors. The Bland-Altman plot showed a mean difference equal to - 0.08 ± 1.63 mmol/mol with 5% of outliers (*i.e.* differences outside the range mean ± 2SD) (Figure 1C).

Similar results were obtained when Cobas c513 was compared with Variant II analyser (Figure 1B and 1D). In this case, the equation of the regression line was: y (HbA_1c_ Variant II, mmol/mol) = 1.00 × (HbA_1c_ Cobas c513, mmol/mol) + 1.57; (95% CI for slope = 0.99 - 1.02 and for intercept = 0.72 - 2.50, indicating a slight constant error). The Bland-Altman plot showed a mean difference equal to - 1.75 ± 1.12 mmol/mol with 2% of outliers.

For these two comparison studies, the cusum test for linearity indicated no significant deviation (P > 0.2) and residual plots presented distribution of differences around fitted regression line without systematic deviation (data not shown).

### Interferences

No analytical interferences of bilirubin and triglycerides were noted for concentrations reaching 352 μmol/L and 20.6 mmol/L, respectively (Table 3). The relative biases, calculated from HbA_1c_ values expressed in mmol/mol, ranged from - 1.4% to + 1.6% for bilirubin, and between - 4.0% to + 0.8% for triglycerides.

**Table 3:** Influence of elevated bilirubin and triglyceride concentrations on HbA_1c_ values obtained by Cobas c513

**Bilirubin (µmol/L)**	**HbA_1c_ Level 1 (mmol/mol)**	**Relative bias (%)**	**HbA_1c_ Level 2 (mmol/mol)**	**Relative bias (%)**
-	36.0	-	62.4	-
88	36.1	+ 0.3	63.4	+ 1.6
176	36.0	0.0	63.2	+ 1.3
264	35.9	- 0.3	63.1	+ 1.1
352	35.5	- 1.4	62.3	- 0.2
**Triglycerides (mmol/L)**				
-	37.7	-	60.4	-
5.2	37.7	0.0	60.7	+ 0.5
10.3	37.6	- 0.3	60.9	+ 0.8
15.5	37.3	- 1.1	59.4	- 1.7
20.6	36.2	- 4.0	59.3	- 1.8
Each value represents the mean of two HbA_1c_ determinations. HbA_1c_ - haemoglobin A_1c_.				

**Figure 2:**
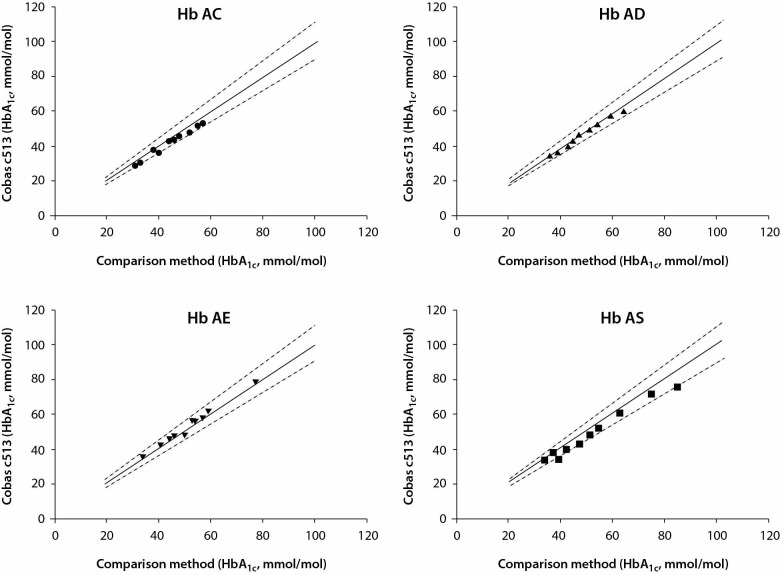
Interference of the most frequent Hb variants. HbA_1c_ values obtained with Cobas c513 analyser for patient samples (N = 10) containing Hb AC, AD, AE or AS variants were compared with HbA_1c_ values obtained by an IFCC-calibrated boronate affinity chromatography method (Premier Hb9210), which is considered a valuable comparison method for the determination of HbA_1c_ in the presence of Hb variants. In each case, the solid grey line represents the regression line calculated using Cobas c513 and Premier Hb9210 results for 40 homozygous HbAA samples whereas the dashed lines represent ± 10% limits with respect to this regression line. HbA_1c_ - haemoglobin A_1c_.

For testing the interference of Hb variants, ten blood samples containing the most frequent variants in their heterozygous forms (Hb AC, AD, AE, AS) were analysed using Cobas c513. HbA_1c_ values were compared with assigned values obtained with a boronate affinity chromatography method, which is recognized to not be affected by the presence of Hb variants. First, the two methods were compared using 40 HbAA samples which were used to establish a linear regression line around which an interval of ± 10% was defined (Figure 2). Haemoglobin variants were considered to cause interference when most of the samples showed HbA_1c_ values outside this range. All tested samples showed values inside this range, regardless of the Hb variant tested, thus, effectively demonstrating the absence of interference of these Hb variants on HbA_1c_ measurement by the Cobas c513 analyser.

### Influence of red blood cell sedimentation

**Figure 3:**
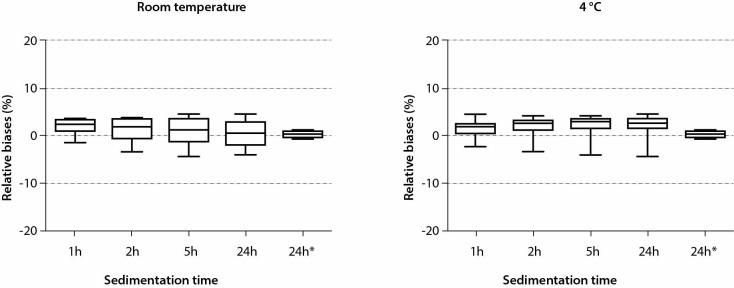
Effect of blood cell sedimentation on HbA_1c_ quantification by Cobas c513. Blood samples (N = 10) were analysed at t0 and then after 1h, 2h, 5h and 24h of sedimentation either at room temperature or at 4°C. At the 24h-sedimentation time, the samples were then agitated and reanalysed (condition 24h*). At each time, the HbA_1c_ values were compared with the values obtained at t0 in order to calculate the relative biases, which are represented as box plots. HbA_1c_ - haemoglobin A_1c_.

The influence of blood cell sedimentation was evaluated by analysing several samples over a 24h sedimentation period. At each time point, the mean relative biases were calculated using the values obtained at time zero as a reference. The results shown in Figure 3 demonstrate that the relative biases did not exceed 5%. The biases were less important when samples have been stored at 4 °C instead of room temperature. The biases were close to 0% when the samples were agitated shortly before analysis at the end of the sedimentation time.

### Usability and ergonomics

The Cobas c513 has a high throughput of up to 400 tests *per* hour on whole blood samples. It allows closed-tube sampling and offers the possibility to work on 10 µL whole blood-capillary straws, which are loaded and remain in the Sarstedt Tina-quant tubes containing 1000 µL of haemolysate. The system requires minimal daily manual maintenance. Moreover, automatic maintenance may be launched during scheduled instrument wake-up, allowing a quicker start-up time. No breakdowns occurred during the evaluation and the availability of tele-maintenance provided training and support on the instrument. Other advantages of this instrument are the availability of automatic identification of tube size and caps, labelling of reagent cassettes by radio-frequency identification, and automatic download of information such as calibrator and QC values, which reduces the need for manual entries.

## Discussion

Haemoglobin A_1c_ is an essential biomarker for monitoring the glycaemic status of patients with diabetes mellitus and is becoming increasingly used for diabetes diagnosis. Its use as a diagnostic tool requires the use of reliable, accurate, and robust analytical methods to avoid misdiagnosis and associated healthcare complications. Fortunately, important advances have emerged in recent years regarding HbA_1c_ assessment, in particular through the global standardization of HbA_1c_ assays and the development of more systematic external quality assurance schemes driven by the IFCC working group and the task force for HbA_1c_ standardization (*14*). This process has improved the homogeneity of HbA_1c_ assessment and reduced the imprecision of the associated methods, thus improving their reliability and in turn facilitating the use of HbA_1c_ for the screening of subjects with diabetes. All methods newly introduced to the market must now be traceable and linked to the IFCC reference method (*7*).

The ever-increasing number of patients with diabetes worldwide is associated with a greater requirement for HbA_1c_ assays. To meet this demand, manufacturers have focused on optimizing their analytical systems in recent years to develop high throughput solutions that retain the technical performances required for reliable HbA_1c_ determination. To this end, Roche Diagnostics have recently developed a new system, the Cobas c513 analyser, which meets the aforementioned criteria and for which the Tina-quant® Third Generation immunoassay has been adapted.

Precision studies of this system yielded promising results, whereby total imprecision CVs did not exceed 1.1% in NGSP units and 1.7% in IFCC units, consistent with the current recommendations for such systems (*6*, *8*). As widely acknowledged in the literature, CVs calculated from values expressed in IFCC units are higher than those obtained from NGSP units (*15*).

The evaluation of accuracy based on the use of samples with IFCC-assigned values showed a mean relative bias of + 1.6% and absolute biases lower than 5 mmol/mol demonstrating a good traceability of this method to the IFCC reference system (*12*). The Cobas c513 analyser was compared with two routine HPLC systems based on the same chromatographic separation principles, comprising an older system with limited throughput (Variant II, dual kit, BioRad Laboratories) and a more recent system (D-100 system, BioRad Laboratories) (*16*). The comparison study showed that both methods were in good agreement with Cobas c513 even though slight proportional and constant errors were found. Moreover, better results were obtained with Variant II system.

We did not observe any significant effect of high concentrations of bilirubin and triglycerides on HbA_1c_ measurement by Cobas c513. Similarly, the presence of the most frequent Hb variants did not significantly modify the observed values, indicating that this method is suitable for HbA_1c_ quantification even in the presence of such variants. We did not assess the impact of labile HbA_1c_ or cHb in this study because, unlike separative methods, immunoassays are generally not affected by these Hb fractions.

Since the analyser does not stir tubes before sampling, we also evaluated the influence of red blood cell sedimentation on the HbA_1c_ assay. The sedimentation test showed that, over a 24h period, HbA_1c_ values remained close to initial values, with relative biases of less than 5%. However, when this test was carried out on tubes kept at 4°C between each measurement, the biases were even lower and came close to zero when the tubes had been shaken after 24h of sedimentation. These results suggest that, even if these differences are not significant, it is advisable to shake the tubes before loading them on the device.

The results of this evaluation are consistent with two other recently published studies, which showed that the Cobas c513 method was comparable to other HbA_1c_ methods and demonstrated that it fulfilled the criteria for becoming a secondary reference measurement procedure (*13*, *17*).

This study has some limitations. For example, the comparison study was carried out using two HPLC systems with similar methodological principles. A comparison with other types of HbA_1c_ assays (*e.g.* capillary electrophoresis or an enzymatic method) could further improve our understanding, although such comparisons have already been described by other authors (*13*, *17*). A further limitation of the present study is that the interference of Hb variants would ideally be analysed in a larger number of samples.

In conclusion, the Cobas c513 analyser exhibits good analytical performance for the measurement of HbA_1c_ at high throughput, making it a reliable system for routine practice in clinical chemistry laboratories performing large-scale HbA_1c_ assays.
